# The Contribution of Various In Vitro Methodologies to Comprehending the Filling Ability of Root Canal Pastes in Primary Teeth

**DOI:** 10.3390/bioengineering10070818

**Published:** 2023-07-09

**Authors:** Claire El Hachem, Jean Claude Abou Chedid, Walid Nehme, Marc Krikor Kaloustian, Nabil Ghosn, Morgane Rabineau, Naji Kharouf, Youssef Haikel, Davide Mancino

**Affiliations:** 1 Department of Pediatric Dentistry, Faculty of Dentistry, Saint Joseph University, Beirut 1107 2180, Lebanon; claire.elhachem@gmail.com (C.E.H.); jcabouchedid@gmail.com (J.C.A.C.); 2 Department of Endodontics, Arthur A. Dugoni School of Dentistry, University of the Pacific, 155 5th Street, San Francisco, CA 94103, USA; wnehme@pacific.edu; 3 Department of Endodontics, Faculty of Dentistry, Saint Joseph University, Beirut 1107 2180, Lebanon; mkaloustian75@gmail.com; 4 Craniofacial Research Laboratory, Faculty of Dental Medicine, Saint Joseph University, Beirut 1107 2180, Lebanon; nabil.ghosn@usj.edu.lb; 5 Faculté de Chirurgie Dentaire, Fédération de Médecine Translationnelle de Strasbourg and Fédération des Matériaux et Nanoscience d’Alsace, Université de Strasbourg, 67000 Strasbourg, France; morgane.rabineau@inserm.fr; 6 Department of Biomaterials and Bioengineering, INSERM UMR_S 1121, Strasbourg University, 67000 Strasbourg, France; youssef.haikel@unistra.fr (Y.H.); mancino@unistra.fr (D.M.); 7 Department of Endodontics, Faculty of Dental Medicine, Strasbourg University, 67000 Strasbourg, France; 8 Pôle de Médecine et Chirurgie Bucco-Dentaire, Hôpital Civil, Hôpitaux Universitaire de Strasbourg, 67000 Strasbourg, France

**Keywords:** calcium silicate material, confocal laser scanning microscope, deciduous tooth, digital microscopy, flowability, micro-CT, pulpectomy primary teeth, root canal filling, SEM, zinc oxide eugenol

## Abstract

A void-free obturation during root canal treatment on primary teeth is currently very difficult to attain. In this study, the pulpectomy filling abilities of Bio-C Pulpecto (Angelus, Basil, Londrina, Paraná, Brazil) and of zinc oxide eugenol, or “ZOE” (DenPro, Prevest, New York, NY, USA), were compared using several in vitro techniques. Therefore, 30 primary anterior teeth were used in the present in vitro study. Analysis of variance (ANOVA), including a multiple comparison procedure (Holm-Sidak method, Dunn’s Method, or Tukey test), was used. On micro-CT, Bio-C Pulpecto exhibited higher void percentages than did ZOE (10.3 ± 3.8%, and 3.5 ± 1.3%), respectively (*p* < 0.05). With digital microscopy, higher total void percentages were found in the BC (13.2 ± 26.7%) group compared to the ZOE (2.7 ± 2.8%) group (*p* < 0.05). With the CLSM, mean tubular penetration depths were higher for Bio-C Pulpecto than for ZOE in all canal thirds (*p* < 0.05). SEM images demonstrated no tags into dentinal tubules in either group throughout the three thirds. Moreover, higher statistically significant flowability was found for Bio-C (2.657 ± 0.06 mm) compared to ZOE (1.8 ± 0.13 mm) (*p* < 0.05). The findings of this study indicate that neither ZOE nor Bio-C Pulpecto appears to meet the criteria for an ideal root canal filling paste for primary teeth. This study laid the groundwork for future research by determining how micro-CT, digital microscopy, SEM, and CLSM contribute to our understanding of the filling process of primary teeth. More thorough research on the mechanism of root canal obturation on primary teeth is required to achieve a long-term successful root canal therapy in young children.

## 1. Introduction

With the introduction of mechanical shaping [1], new irrigation protocols [2], and new filling materials and techniques [3], root canal therapy for primary teeth is rapidly developing. Success is still not always assured, and in the present clinical pulpectomy practice, attaining a void-free root canal obturation is difficult [4]. It is crucial to choose the filling paste with the best biological, mechanical, and physicochemical properties to obtain dense 3D obturation, avoid shrinking or irritating the periapical tissues, and ensure that the filling paste resorbs concurrently with the roots without damaging the underlying tooth successor [5].

Zinc oxide eugenol (ZOE powder and liquid), calcium hydroxide paste alone or mixed with iodoform combined with rotary Lentulo spirals, premixed syringes, and endodontic pluggers/reamers were suggested to enhance the quality of obturation on primary teeth [6,7]. However, there is currently still no agreement on the best root canal filling material for primary teeth, and each substance has disadvantages. ZOE sets into a thick mass that resists resorption, may irritate periapical tissues, and can cause deviation of the permanent tooth bud [8]. Calcium hydroxide-based materials may result in intracanal and external resorption, resulting in long-term failure of the treatment [9].

New endodontic forms of cement, called bioceramics, have been gaining popularity due to their physicochemical and biological characteristics, such as their alkaline pH, shrink-free property, chemical stability in the biological environment, and biocompatibility [10,11]. In permanent teeth, these bioactive materials, which exhibit biological activity [12], have many clinical indications such as pulpotomies, pulp capping, resorption, perforation repair, and root canal fillings [13,14,15,16]. Pediatric dentists have recently endorsed them as well. The first resorbable bioceramic root canal filling for primary teeth is called Bio-C Pulpecto (Angelus, Basil, Londrina, Paraná, Brazil). It is made up of silicon dioxide, calcium tungstate, titanium oxide, ester glycol salicylate, toluene sulphonamide, and calcium silicate [5].

To assess the quality of a root canal filling, there are various in vitro methods, and each method enables an understanding of a certain aspect of the obturation. The most precise non-invasive imaging method that has received widespread support from studies and enables a quantitative assessment of internal structural changes in root canal morphology is micro-Computed Tomography (µCt) [17,18]. Microscopes, despite being destructive, are essential for understanding the mechanism of endodontic materials’ penetration into dentinal tubules; options include using a confocal laser scanning microscope (CLSM) [19] and/or scanning electron microscope (SEM) [20]. CLSM reveals information about the sealer penetration and distribution inside the dentinal tubules of root canal walls by including a fluorescent dye marker with the pastes, while SEM allows evaluation of sealer adaptation with root canal walls and marginal gaps. In primary dentition, there are very few publications that integrate several in vitro evaluation techniques to assess the ability of root canal filling pastes used [21,22].

In the pediatric endodontic literature, there is an agreement about the difficulty of obtaining a void-free obturation with long-term success. In a meta-analysis totalizing 263 teeth, the authors stated that there is currently no scientific evidence of the superiority of any one root canal filling material for endodontic treatment of necrotic primary teeth [23]. Studies confirmed that a good hermetic seal with minimum voids is directly related to the material’s capacity to adhere to the walls of the root canal and the method used to deliver this material into the root canal [6,24]. It was also reported that primary teeth filling pastes lead to overfilled canals and resorption within the root [25].

This study’s main objective was to evaluate the pulpectomy filling abilities of zinc oxide eugenol and Bio-C Pulpecto. For each material, the percentage of voids/total filling, the flowability, the penetration depths, and the dentinal tags were assessed utilizing micro-CT, CLSM, digital microscopy, and SEM. The goal was to evaluate the data from each procedure and classify them so that clinicians could fully understand all facets of root canal filling on primary teeth. The null hypothesis is that there is no difference in the filling ability of zinc oxide eugenol and Bio-C Pulpecto when assessed with micro-CT, CLSM, digital microscopy, and SEM.

## 2. Materials and Methods

### 2.1. Teeth Selection

The ethics committee of the Saint Joseph University of Beirut, Lebanon (USJ-2019-237) approved this study. One hundred primary anterior teeth with minimal root resorption, belonging to children aged 3 to 6 and extracted for reasons unrelated to this study as part of treatment plans at the University of X’s Department of Pediatric Dentistry, were collected and kept in formocresol 0.1%. Teeth with previous pulpotomy or pulpectomy, internal resorption, and advanced root resorption were excluded after inspection under an operating microscope. Therefore, 30 primary anterior teeth were included in this study. Using the IBM SPSS statistics software (version 27.0), the sample size was calculated. To ensure more than 80% power and an alpha error probability of 0.05, two groups of 15 canals each were formed.

### 2.2. Teeth Preparation

Patency was verified with a size 10 K-file (Dentsply Sirona, Ballaigues, Switzerland) following access cavity preparation. A diamond disc (Kerr Dental, Bioggio, Switzerland) was used to section the crowns to standardize the root length at 12 mm, and the working length (WL) was determined, 1 mm short of the apical foramen, with a size 15 K-file (Dentsply Sirona, Ballaigues, Switzerland). For the shaping, R-motion^®^ 21 mm file (30/0.04) (FKG Dentaire SA, La Chaux-de-Fonds, Switzerland) was used to prepare all the canals. Using a 30G side-vented needle (NaviTip, Ultradent), 12 mL of 1% NaOCl was flushed inside the canals. Mechanical activation of the irrigant with XP-endo Finisher (FKG) operated at 1000 rpm, as suggested by the manufacturer, was carried out for 30 s in all canals; the tip was placed 1 mm short of the WL without binding. After drying the canals with sterile paper points, 1 mL of 17% EDTA was injected and left for 1 min inside the canals. Following the same protocol, EDTA was activated. For the final irrigation, 3 mL of saline was used. Canals were dried with paper points.

### 2.3. Root Canal Obturation

According to the filling materials, the teeth were divided into 2 groups. Furthermore, to perform analysis under CLSM, each filling paste was manually labeled with rhodamine B powder (Sigma-Aldrich, St. Louis, MO, USA) to an approximate concentration of 0.1% to provide fluorescence and allow confocal laser microscopy assessment [26]. 

Group 1: Zinc oxide eugenol was used in the form of powder liquid and was mixed in a ration 2:1 to obtain a creamy consistency. The labeled cement was inserted into the canal 1 mm short of the WL, with a size 30 Lentulo spiral (Dentsply Maillefer, Ballaigues, Switzerland) used for at least five seconds inside the canal in little pecking motions. 

Group 2: Bio-C Pulpecto (BC), a premixed bioceramic material, was emptied on a plexiglass, marked with the fluorescent dye, refilled into the syringe, and injected directly into the canals. 

To validate the quality of the filling in terms of length and density, a buccolingual and a distomesial digital radiograph were taken. None of the teeth exhibited a poor quality of obturation; therefore, none were discarded.

Afterward, the access cavity was sealed with Teflon tape and reinforced zinc oxide eugenol (Intermediate Restorative Material, IRM; Dentsply Sirona, Charlotte, NC, USA). The teeth were then incubated in the dark in a container (Memmert GmbH, Büchenbach, Germany) at 37 °C for 14 days with full saturated humidity to ensure the final setting.

### 2.4. Micro CT Scanning

For tooth imaging, a micro-CT Platform (EA2496, Montrouge, France) was used to investigate the 30 teeth. Each tooth was individually scanned with a micro-CT scanner (Quantum FX; PerkinElmer Health Sciences; Hopkinton) to measure the void volume (µm^3^) in each third in order to assess the filling percentage and voids in the coronal, middle, and apical thirds. The field of view was set at 10 mm to acquire 3D images with an isotropic resolution of 20 µm. Acquisition settings were 160 kV, 90 mA, and 360° scanning rotation.

DICOM data were imported into 3D Slicer 5.1 software. The following semi-automated threshold-based segmentations were realized:

- Complete root with filling
- Complete filling with voids
- Filling without voids

Boolean operations were performed to get the following 3D segmentation:

- C: Canal (filling + voids)
- F: Filling without voids
- V: Voids

The software’s Models module transformed the aforementioned 3D segmentations into 3D models and automatically measured each model’s volume.

The following calculation was used to determine the percentage of voids in the obturation:

V/C × 100

To calculate the percentage of filling and voids in the coronal, middle, and apical sections, the total length of the canal was measured, divided by 3, and then 2 custom plane sections perpendicular to the long axis of the canal were realized to separate the coronal, middle, and apical models at equidistant lengths. C, F, and V were calculated for each part as follows [27]:

- Cc, Fc, and Vc for the coronal part
- Cm, Fm, and Vm for the middle part
- Ca, Fa, and Va for the apical part

The same formula was used to calculate the percentage of voids in canal thirds [27]. 

**Figure 1:**
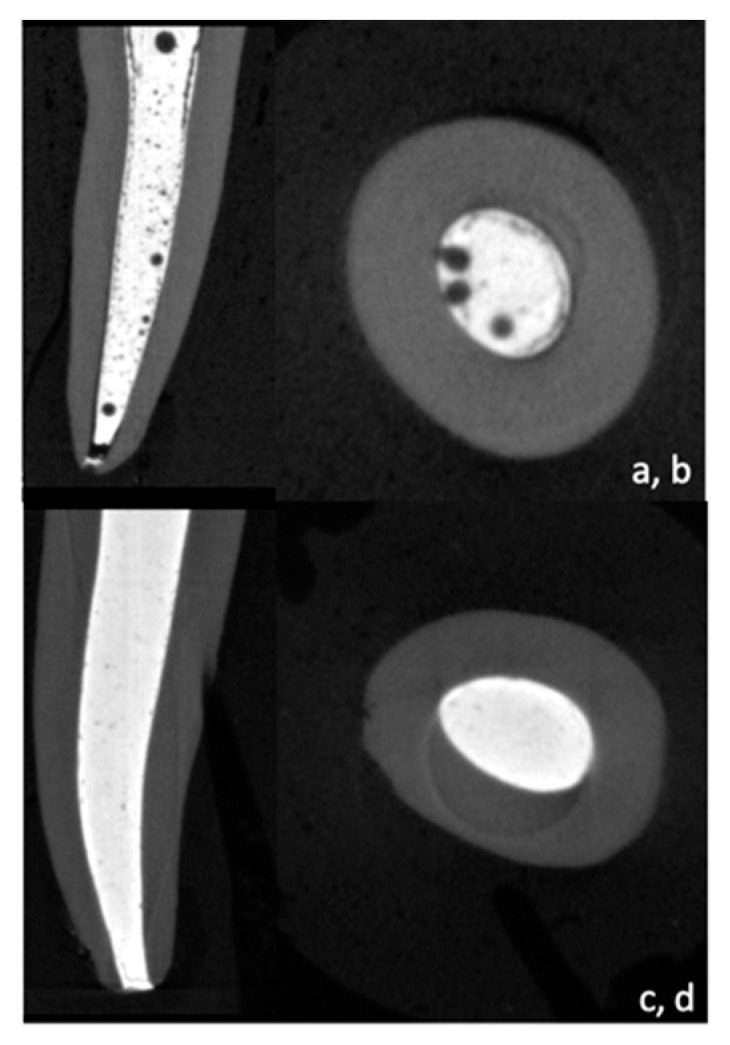
Micro-CT cross sections and reconstructed 3D image showing Bio-C Pulpecto (**a**,**b**) and ZOE (**c**,**d**).

The percentage of filling and voids was evaluated using a threshold method and 3D models in the coronal, middle, and apical sections (Figure 1). 

### 2.5. Sectioning

The root canals of the 30 teeth were cross-sectioned at 1 mm and 5 mm from the root apex using a diamond disk (Buehler, Lake Bluff, IL, USA) and a slow speed (25,000 rpm) handpiece. After mounting the specimens onto glass slides, the coronal surface was polished with sandpapers of 600, 1200, 2400, and 4000-grit silicon carbide paper (Escil, Chassieu, France) under running water. The sample examined by confocal laser microscopy has a thickness of 2 mm [19].

### 2.6. Digital Microscopy Observations

**Figure 2:**
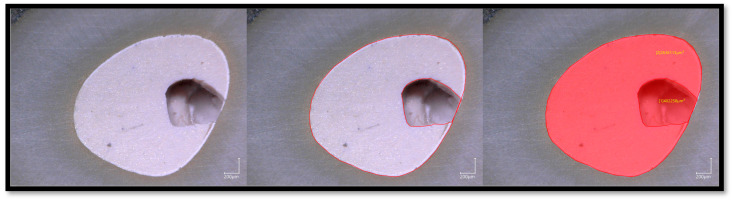
Methodology for measuring the area of filling materials and voids with the VHX-5000 program.

Specimen polished surfaces (*n* = 90, three surfaces for each tooth) were first examined under a digital microscope VHX-5000 (KEYENCE, Osaka, Japan), and one image was captured for each specimen at 100× magnification. The micrographs were coded by an expert examiner who was not involved in the experiment, displaying the canal wall surface of both groups at the coronal, middle, and apical thirds, for blinded analysis using the VHX-5000 software (KEYENCE, Osaka, Japan) to measure the total area of the filling materials and of the internal and external voids in µm^3^ following a previous study [28] (Figure 2). After that, the percentages of voids were calculated and statistically analyzed.

### 2.7. Confocal Laser Scanning Microscopy Analysis

Confocal laser scanning microscope (Zeiss, LSM 710, Göttingen, Germany) with an objective 10× Plan NeoFluor and a 514 nm excitation wavelength compatible with rhodamine dye was used to examine all the canals (*n* = 90, three surfaces for each tooth). The entire dentinal tubule penetration area was determined using ImageJ software (NIH). The deepest penetration from the canal wall to the point of maximum sealer penetration was calculated using ImageJ software. Each measurement was performed twice to assure accuracy and reproducibility. The penetration depths at 8 circumferential sites were averaged to obtain the mean sealer penetration depths in µm with the highest degree of accuracy at the coronal, middle, and apical sections for Bio-C Pulpecto and ZOE. 

### 2.8. Scanning Electron Microscope Observations

From each group, six samples, including the three thirds, were chosen to closely inspect the areas where filling paste and dentin met. To observe the materials’ tags into the dentinal tubules, the polished surfaces were etched with 37% phosphoric acid for 10 s and immersed in 2.5% NaOCl for 3 min [29]. After that, the specimens were dehydrated in a graded series of ethanol solutions (50, 70, 95, and 100%) for 10 min each before being coated with a gold-palladium alloy (20/80 weight percent) using Hummer JR sputtering equipment (Technics, Rocklin, CA, USA). The produced samples were examined using a Quanta 250 FEG scanning electron microscope (FEI Company, Eindhoven, The Netherlands) with an electron acceleration voltage of 10 kV and a magnification of 100–4000 [30]. These samples were examined under SEM to verify the findings obtained using CLSM and digital microscopy.

### 2.9. Flow Test

Additionally, a flow test was conducted using the method outlined in ISO 6876/2012: 50 µL of each mixed sealer (in triplicate) was dispensed on a separate glass plate (40 × 40 × 5 mm). A second glass plate was carefully placed on top of the sealer after 3 min of mixing. Then, a weight of 100 g was applied centrally on top of the second glass plate. After 10 min, using a digital caliper (Dexter, Elkhart, IN, USA), the compressed sealer’s maximum and lowest diameters were measured. The test was repeated to determine the mean diameter if there was a discrepancy of greater than 1 mm between the two measurements [31].

### 2.10. Statistical Analysis 

SigmaPlot (release 11.2, Systat Software, Inc., San Jose, CA, USA) was used for statistical analysis. The Shapiro–Wilk test was used to verify the normality of the data in all groups. Analysis of variance (ANOVA), including a multiple comparison procedure (Holm-Sidak method, Dunn’s Method, or Tukey test), was used to determine whether significant differences existed in the void evaluations between the different techniques and materials. A statistical significance level of *p* = 0.05 was adopted in all tests.

## 3. Results 

### 3.1. Micro-CT

When comparing overall void percentages, Bio-C Pulpecto exhibited higher void percentages compared to ZOE (10.3 ± 3.8%, and 3.5 ± 1.3%), respectively (*p* < 0.001). Additionally, the apical third of the ZOE group had higher void percentages than the coronal and middle thirds (*p* = 0.006), but there was no statistically significant difference between the middle and coronal thirds (*p* > 0.05). There were no statistically significant differences between the three thirds of the Bio-C Pulpecto group (*p* = 0.192) (Table 1).

### 3.2. Digital Microscopy

**Figure 3:**
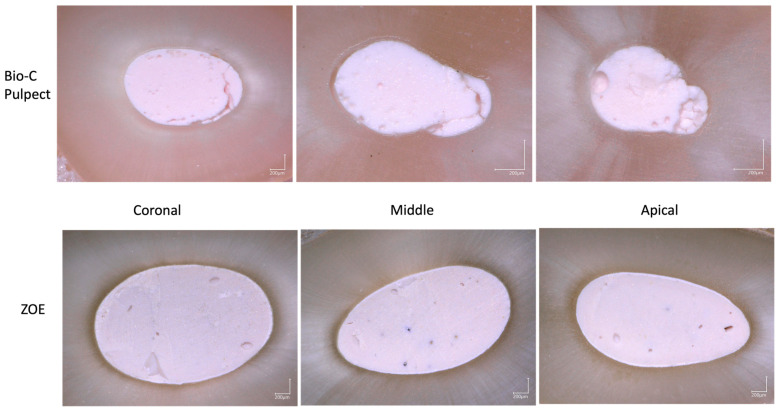
Digital microscopy images for Bio-C Pulpecto and ZOE in the coronal, middle, and apical thirds.

The same tendency was found for the results of Keyence compared to micro-CT outcomes. Higher total void percentages were found in the BC group compared to the ZOE group in the coronal, middle, and apical thirds (Table 2). BC demonstrated higher void percentages compared to ZOE at the apical (*p* < 0.001), middle (*p* = 0.002), and coronal (*p* = 0.019) thirds (Table 2 and Figure 3).

### 3.3. Confocal Laser Scanning Microscope

Mean tubular penetration depths were higher for Bio-C Pulpecto than for ZOE in the coronal (277 ± 124 µm and 122 ± 62 µm), middle (247 ± 118 µm and 112 ± 55 µm), and apical thirds (218 ± 114 µm and 102 ± 52 µm), respectively (Table 3).

**Figure 4:**
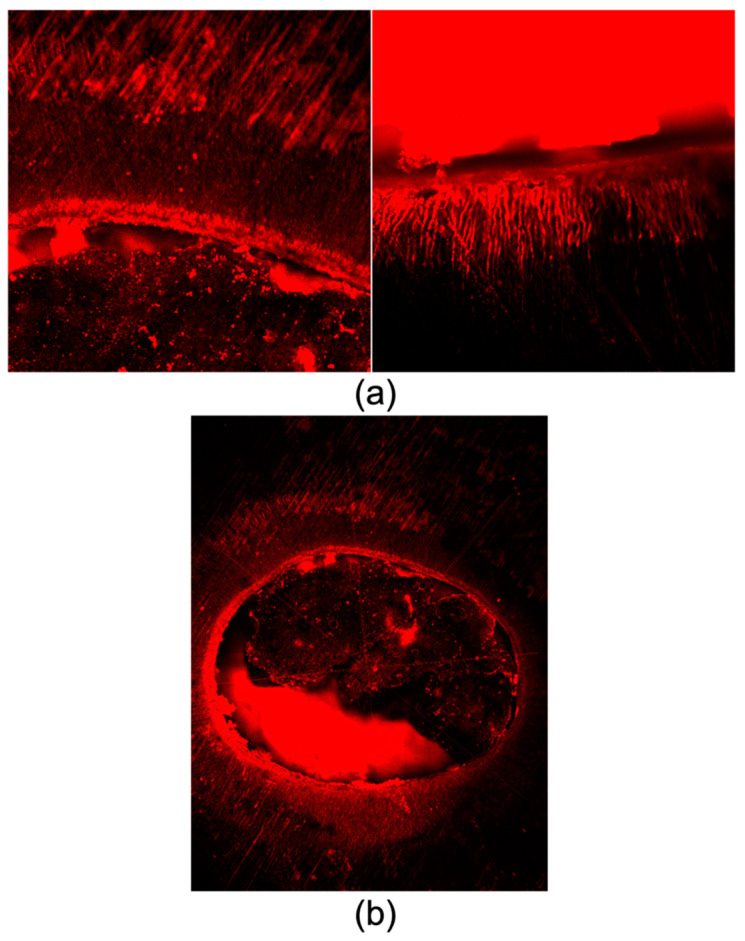
(**a**) ZOE and Bio-C Pulpecto penetration into dentinal tubules; (**b**) methodology for calculating mean penetration depth into dentinal tubules.

In addition, in the ZOE and BC groups, statistically higher material infiltrations values were observed in the coronal third compared to the apical third ((*p* = 0.012) and (*p* < 0.001), respectively), while no statistically significant differences were found between the apical/middle and coronal/middle (*p* > 0.05) (Figure 4).

### 3.4. Scanning Electron Microscope (SEM vs. CLSM)

**Figure 5:**
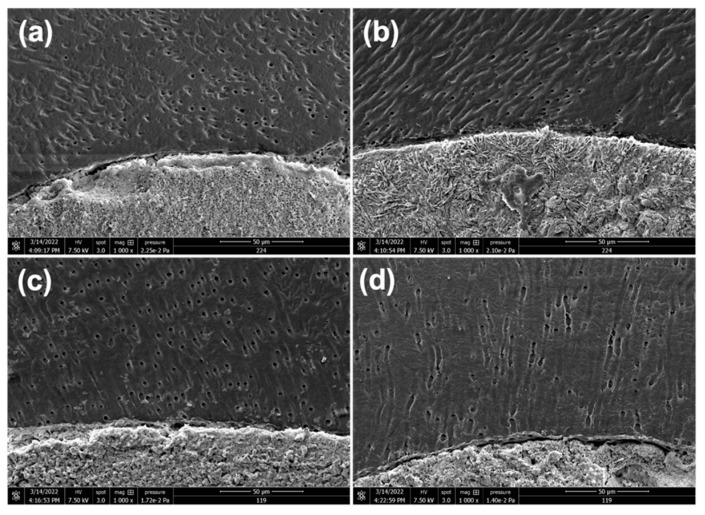
Representative SEM images showing no tags into dentinal tubules for ZOE in the middle (**a**) and apical thirds (**b**) or for Bio-C Pulpecto in the middle (**c**) and apical thirds (**d**).

In contrast with the confocal results, SEM images demonstrated no tags into dentinal tubules in either group throughout the three thirds (Figure 5). 

### 3.5. Flow Test 

Higher statistically significant flowability was found for Bio-C (2.657 ± 0.06 mm) compared to ZOE (1.8 ± 0.13 mm) (*p* < 0.001).

## 4. Discussion

Root canal treatment for primary teeth has seen significant development recently [32]. The best root canal filling material and procedure are still up for debate, though. Despite its disadvantages and potential toxicity, zinc oxide eugenol is nonetheless utilized routinely and with great success [8]. Nonetheless, the hunt for biocompatible and bioactive materials has led to the recent development of bioceramics, such as the non-setting Bio-C Pulpecto for primary teeth [5]. In this study, we used a variety of in vitro evaluation techniques to examine the filling ability of zinc oxide eugenol (ZOE) and Bio-C Pulpecto. Since there was a significant difference between ZOE and Bio-C Pulpecto according to all in vitro evaluation techniques, the null hypothesis was partially rejected (*p* < 0.05). 

The goal of this study was to assess and categorize the data obtained from micro-CT, digital microscopy, CLSM, and SEM so that clinicians could comprehend every aspect of root canal filling on primary teeth. The novelty of this study consists of its combination of numerous in vitro methodologies to evaluate the filling capabilities of root canal pastes used on primary teeth and its choice of the filling material, namely a bioceramic created exclusively for primary teeth. Therefore, each technique would enable comprehension of a certain component of the obturation and provide never-before-published data.

The first methodology adopted in this study to compare the filling ability of ZOE and Bio-C Pulpecto was micro-CT imaging. Micro-CT is used to analyze tooth structure objectively, allowing for quantitative and qualitative image analysis [33,34]. Additionally, it enables the accurate reconstruction of 3D models and can distinguish between tooth structures, voids, and obturation materials [35]. Both materials produced voids in all canal thirds, with Bio-C Pulpecto revealing higher void percentages than ZOE (10.3 ± 3.8%, and 3.5 ± 1.3%). This was per the results of numerous studies, regardless of the filling material, that agree on the difficulty of achieving a void-free obturation due to the complex root canal anatomy of human teeth [36,37]. 

In addition, in primary dentition, the obturation relies exclusively on a resorbable filling paste, without the support of a gutta-percha cone, which renders the 3D obturation even more difficult, especially with the abundance of lateral canals, isthmus, and canal curvatures [32,38]. In one of the few previous micro-CT studies on primary teeth filling, the authors suggested that using a syringe to inject the paste produced fewer voids than using a lentulo spiral [7]. This could explain why, in this study, there was an increase in apical voids for the ZOE group (*p* < 0.05), whereas there was no discernible difference between coronal, middle, and apical thirds for the Bio-C Pulpecto group (*p* > 0.05). In fact, in an attempt to enhance the quality of root canal obturation on primary teeth and decrease the void volume, some authors proposed ultrasonic activation of the filling paste for a better infiltration in the intricate primary teeth anatomy [39]. More micro-CT studies should be conducted to find the most efficient filling technique for primary teeth. 

The samples were then sectioned and further analyzed with different microscopes. 

Using the digital microscope, the filling volume and voids were quantified and measured in the coronal, middle, and apical sections for both groups. The results corroborate the micro-CT findings showing higher total void percentages in Bio-C Pulpecto (13.2 ± 26.7%) compared to ZOE (2.7 ± 2.8%) (*p* < 0.05). However, in contrast to micro-CT, no statistically significant differences were found between the three thirds for both materials (*p* > 0.05), while micro-CT demonstrated a significant difference between the three thirds of the ZOE group. This could be related to the high resolution and precision provided by micro-CT, which can detect more details, as well as to the ability of micro-CT to investigate the void in volume (3D) while the digital microscope could be used to investigate only in the slices (one section for each third), which is a limitation of the digital microscope in void investigation [31]. The two methodologies allow for quantitative evaluation of total filling percentage, voids, and detection of flaws in the bulk filling of root canal pastes for primary teeth. Therefore, both techniques showed the same tendency for both materials with higher detection for the micro-CT method.

A fluorescent rhodamine marker mixed with pastes was used to visualize the penetration and distribution of the sealers within the dentinal tubules of root canal walls using a CLSM [19]. Mean tubular penetration depths were higher for Bio-C Pulpecto than for ZOE in all canal thirds. This could be attributed to the physicochemical properties of Bio-C Pulpecto, its high contact angle, and its solubility, presumably allowing it to easily diffuse into the dentinal tubules [5]. This finding was also verified by conducting a flowability test that showed higher mean values for Bio-C Pulpecto than for ZOE. Studies agree that the penetration depth inside dentinal tubules is directly related to the properties of the materials such as setting time and flowability [40]. Moreover, under CLSM, for both materials, the depth of sealer penetration into dentinal tubules decreased from the coronal to the apical part (*p* < 0.05). This could be attributed to the obturation technique since both the lentulo spiral and the pressure syringe techniques lead to voids in the apical part [39]. This may also be accounted for by the apical region’s lower density and diameter of dentinal tubules [41]. Future studies should focus on developing filling pastes with enhanced abilities to penetrate the dentinal tubule, encapsulate the bacteria inside, and favor interaction between the material and the dentinal fluid. 

To visualize the adaptation of filling pastes to canal walls and marginal gaps and to detect the material tags in dentinal tubules, some samples were further observed under SEM. The intermolecular surface energy and cleanliness of the dentin, as well as the surface tension and wetting capacity of the sealer, all interact to determine the degree of adhesion [30]. The retention of filling material by root canal walls is improved by sealer plugs placed into the dentinal tubules because they mechanically interlock [42]. In the current study, in contrast to CLSM infiltration images, SEM images demonstrated no tags into dentinal tubules in either group throughout the three thirds. This could be due to the irrigation protocol being insufficient in eliminating the debris and smear layer and also to the consistency of the paste and the filling technique [30]. This could also be related to the detachment of the fluorescent dye from the filling paste, which gives the fake impression of an infiltrated dentinal tubule. Some authors even concluded that bioceramic sealers should not be utilized in conjunction with Rhodamine for CLSM assessment after it was reported that the kind of fluorophore changes the calcium silicate sealers’ performance when using CLSM [43]. In addition, we can hypothesize that the several in vitro steps that were performed to prepare the samples for SEM observations, including sectioning, polishing, and chemical preparations to eliminate the smear layer and to dry the samples, could alter this observation and could dissolve the material tags.

Overall, the findings of this study indicate that neither ZOE nor Bio-C Pulpecto appears to meet the criteria for an ideal root canal filling paste for primary teeth. The purpose of this study was to create the groundwork for future research by determining how micro-CT, digital microscopy, SEM, and CLSM contribute to our understanding of the filling process. In this study, ZOE was superior to Bio-C Pulpecto according to micro-CT and digital microscopy, whereas CLSM and SEM produced contradictory findings, with CLSM indicating tubular infiltration for both pastes and SEM disproving this claim by demonstrating no dentinal tags for either group. It should be nonetheless noted that microscopes are invasive and allow only partial evaluation of root fillings and that some may create irreversible damage to the specimens [44,45]. These factors might lead to inaccuracies because some filling material might be lost during sample preparation [46]. 

To create better materials for pediatric endodontics, additional evidence-based research is urgently required to completely understand whether the issue is with the filling pastes, the filling technique, or most likely both, and how to fix it. More thorough research on the mechanism of root canal obturation on primary teeth is required. Numerous studies indicate that for the time being, it is impossible to obturate primary teeth in a confined, dense 3D space, putting the effectiveness of root canal therapy on primary teeth in jeopardy [47,48,49].

## 5. Conclusions

Current primary tooth filling pastes, including ZOE or Bio-C Pulpecto, do not meet the criteria for the ideal root canal filling material. Micro-CT and digital microscopy revealed that ZOE was superior to Bio-C Pulpecto; however, CLSM and SEM provided inconsistent results, with CLSM showing tubular infiltration for both pastes and SEM refuting this assertion by showing no dentinal tags for either group. The qualities and methods of filling materials should be the focus of future research. Most significantly, the studies need to find a way to improve the effectiveness of root canal filling pastes for primary teeth using all current in vitro imaging or microscopic techniques.

## Figures and Tables

**Table 1:** Mean and standard deviations of void percentages in ZOE and BC groups after micro-CT analysis. Zinc oxide eugenol (ZOE), Bio-C Pulpecto (BC), coronal (C), middle (M), and apical (A).

	Coronal (%)	Middle (%)	Apical (%)	Statistical Analysis
ZOE	2.7 ± 1.3	2.3 ± 1.8	6.7 ± 4.9	A > C, A > M
BC	7.5 ± 4	8.9 ± 7.7	17.2 ± 14.8	No
Statistical analysis	*p* < 0.001	r = 0.002	*p* = 0.049	

**Table 2:** Mean and standard deviations of void percentages in ZOE and BC groups after digital microscope analysis. Zinc oxide eugenol (ZOE), Bio-C Pulpecto (BC), coronal (C), middle (M), and apical (A).

	Coronal (%)		Middle (%)		Apical (%)	
	Close	Open	Close	Open	Close	Open
ZOE	2.6 ± 2.1	0.2 ± 0.4	1.6 ± 1.8	0.3 ± 0.8	1.48 ± 1.77	1.7 ± 3.7
BC	4.4 ± 7.4	5 ± 7.7	2.2 ±1.6	4.8 ± 5	13.7 ± 32.7	9.4 ± 13.6
Statistical analysis	*p* = 0.019		*p* = 0.002		*p* < 0.001	

**Table 3:** Mean and standard deviations of void percentages in ZOE and BC groups after confocal microscope analysis. Zinc oxide eugenol (ZOE); Bio-C Pulpecto (BC), Coronal (C); Middle (M) and Apical (A).

	Coronal (%)	Middle (%)	Apical (%)	Statistical Analysis
ZOE	122 ± 62	112 ± 55	102 ± 52	C > A
BC	277 ± 124	247 ± 118	218 ± 114	C > A
Statistical analysis	*p* < 0.001	*p* < 0.001	*p* < 0.001	

## Data Availability

Not applicable.
